# Interactions of Alginate-Deferoxamine Conjugates With Blood Components and Their Antioxidation in the Hemoglobin Oxidation Model

**DOI:** 10.3389/fbioe.2020.00053

**Published:** 2020-02-11

**Authors:** Tong Sun, Xi Guo, Rui Zhong, Chengwei Wang, Hao Liu, Hao Li, Lu Ma, Junwen Guan, Chao You, Meng Tian

**Affiliations:** ^1^Neurosurgery Research Laboratory, National Clinical Research Center for Geriatrics, West China Hospital, Sichuan University, Chengdu, China; ^2^Department of Neurosurgery, West China Hospital, Sichuan University, Chengdu, China; ^3^Institute of Blood Transfusion, Chinese Academy of Medical Sciences and Peking Union Medical College, Chengdu, China; ^4^Department of Integrated Traditional Chinese and Western Medicine, West China Hospital, Sichuan University, Chengdu, China; ^5^West China Brain Research Centre, West China Hospital, Sichuan University, Chengdu, China

**Keywords:** deferoxamine, blood components, antioxidation, alginate, conjugates

## Abstract

While deferoxamine (DFO) has long been used as an FDA-approved iron chelator, its proangiogenesis ability attracts increasing number of research interests. To address its drawbacks such as short plasma half-life and toxicity, polymeric conjugated strategy has been proposed and shown superiority. Owing to intravenous injection and application in blood-related conditions, however, the blood interactions and antioxidation of the DFO-conjugates and the mechanisms underlying these outcomes remain to be elucidated. In this regard, incubating with three different molecular-weight (MW) alginate-DFO conjugates (ADs) red blood cells (RBCs), coagulation system, complement and platelet were investigated. To prove the antioxidant activity of ADs, we used hemoglobin oxidation model *in vitro*. ADs did not cause RBCs hemolysis while reversible aggregation and normal deformability ability were observed. However, the coagulation time, particularly APTT and TT, were significantly prolonged in a dose-dependent manner, and fibrinogen was dramatically decreased, suggesting ADs could dominantly inhibit the intrinsic pathways in the process of coagulation. The dose-dependent anticoagulation might be related with the functional groups along the alginate chains. The complements, C3a and C5a, were activated by ADs in a dose-dependent manner through alternative pathway. For platelet, ADs slightly suppressed the activation and aggregation at low concentration. Based on above results, the cross-talking among coagulation, complement and platelet induced by ADs was proposed. The antioxidation of ADs through iron chelation was proved and the antioxidant activity was shown in a MW-dependent manner.

## Introduction

Deferoxamine (DFO) has long been used as an FDA-approved intravenously injected iron chelator in clinic for half a century in the treatment of iron overload diseases, while in the recent years, a great deal of attention was given to the important applications of DFO in the growing field of tissue regeneration as a result of its unique properties to inhibit inflammation and promote vascularization (Guo et al., 2019; Holden and Nair, 2019). In the early studies, DFO was proved to stimulate angiogenesis and neovascularization in the ischemia model of sheep and rabbit, and since then, increasing number of studies attempt to elucidate the related mechanisms (Chekanov et al., 2003). To date, it is widely accepted that DFO is contributed to the prevention of degradation of hypoxia-inducible factor-1 alpha (HIF-1α), an oxygen-sensitive molecule to upregulate the expression of vascular endothelial growth factor (VEGF) (Wu et al., 2010). In the meanwhile, some studies indicated that angiogenesis induced by DFO was closely associated with the property of antioxidation, as a secondary effect (Jiang et al., 2014). Despite the dispute, the ability of angiogenesis in addition to the antioxidation make it serve as a promising candidate for various biomedical use.

However, the application of DFO is still of much limitation owing to its drawbacks, like other small molecular drugs, including short plasma half-life and toxicity (Cassinerio et al., 2014). In this regard, polymer-drug conjugation strategy is appropriate for DFO to overcome these obstacles (Yan et al., 2016). Polymer-drug conjugates belonging to nano-sized drug delivery system attracts great research interest and are becoming established as a shining platform for drug delivery due to the reasons that the conjugation strategy deeply changes the behavior of the corresponding parent drugs and offers them many benefits, including high drug-loading, prolonged circulatory half-life, and reduced toxicity (Zhou et al., 2015; Ding et al., 2019a; Jesus et al., 2019; Macha et al., 2019; Qi et al., 2019). However, the therapeutic efficiency in clinic is not as expected, e.g., less than 10% of a systemically administered dose accumulates within the lesion, and in some cases there is no significant improvement for patient survival rate such as FDA approved doxorubicin HCl liposome (DOXIL) for anticancer (Zhang et al., 2013). Hence, the reasons why these conjugates with fine-tuned structures do not function as intended need to be elucidated in order to advance the therapy in clinic (Feng et al., 2019).

One of the most factors that contributes to this discrepancy is the interactions that exist between intravenously injected conjugates and the blood, since these interactions may change the target and transport capabilities of conjugates, thus determining the fate and the final therapeutic efficiency of conjugates in body (Lazarovits et al., 2015; Ding et al., 2019b). To develop solutions to these barriers, it is crucial to study the blood-conjugates interactions, e.g., RBCs, coagulation function, protein adsorption, complement system and platelets, which depended on the properties of conjugates such as structure, molecular weight (MW), and the functional groups along the chains (Wu et al., 2017). Besides, several pathways and mechanisms underlying these interactions suggest that some cross-talking among them has to be involved (Fornaguera et al., 2015). For instance, protein adsorption is deemed as the initial event in blood-conjugates interactions (Kenry et al., 2016), leading to the activation of coagulation cascade that can contribute significantly to both of complement system and platelet through certain coagulation enzymes (Liu et al., 2014). Nevertheless, the interplay among coagulation, platelet and complement is still of much limitation to be fully understood in the process of thrombus formation and inflammation response (Speth et al., 2015).

Actually, some studies had proven that conjugation of DFO into polymer carriers, such as hydroxyethyl starch, dextran, dialdehyde cellulose, nanoparticles, and polyethylene glycol copolymer, significantly prolonged the half-life comparing to the free of DFO (Tian et al., 2016a). To better of our knowledge, there is no report on systematic study of the DFO-based blood-conjugates interactions. Alginate, consisting of α-L-guluronate (G unit) and β-D-mannuronate (M unit), has long been used in delivery systems and in terms of the interactions with blood, alginate could induce aggregation of RBCs, allowing it be used as a viscosity modifier for blood substitutes. Contributing to the versatile functional groups, e.g., carboxyl and hydroxyl, along the molecular chains, alginate is also suggested to have effects on the protein adsorption and complement activation (Zhao et al., 2010; Şen, 2011). In this light, the interactions of alginate-DFO conjugates is rather crucial to quest the potential mechanism. On the other hand, alginate has its intrinsic antioxidant activity due to the reductive ability of residues along the molecular chains, which may benefit to protect hemoglobin from oxidation in a different manner comparable to DFO. However, the polymer drug carrier also leads to steric hindrance for DFO cheating, resulting in iron binding occurring at a slower rate, depending on the density and location of conjugated DFO molecules.

**FIGURE 1:**
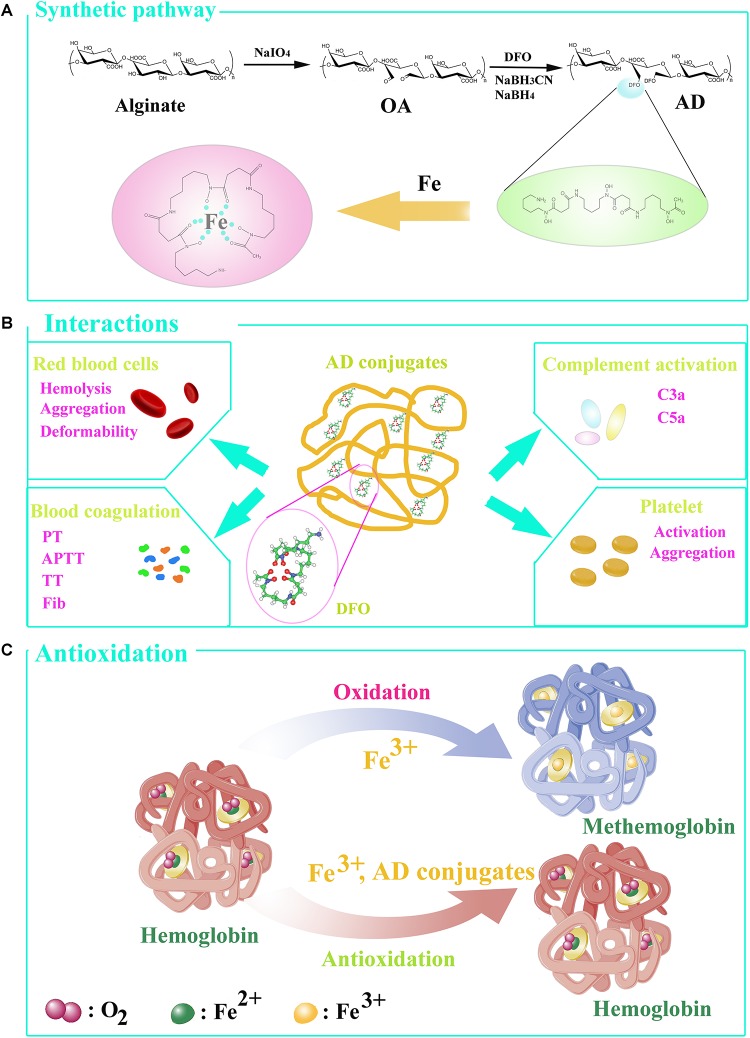
**(A)** The synthesis of conjugates. **(B)** The interactions of ADs with blood in vitro. **(C)** Hemoglobin oxidation model. Under normal circumstance, the hemoglobin converts to oxyhemoglobin in ferrous state (Fe^2+^), able to bind and transport oxygen, and methemoglobin in the presence of ferric iron (Fe^3+^). AD conjugates can bind to Fe^3+^ so that oxyhemoglobin was generated to transport oxygen.

In this work, alginate was chosen as a polymer carrier to prepare a series of alginate-DFO conjugates (ADs) with various MW and study their interactions with blood. We hypothesize that the molecular weight and functional groups along the alginate chains have specific interactions with blood. To address this hypothesis, a series of ADs with different MW were synthesized (Figure 1A). Incubating with ADs, RBCs, coagulation system, complement, and platelet, were investigated (Figure 1B). Specifically, the RBCs’ properties including hemolysis, aggregation and deformability, were determined. Blood coagulation time including prothrombin time (PT), activated partial thromboplastin time (APTT) and thrombin time (TT), and the concentration of fibrinogen (Fib), were tested. The concentration of completement 3a (C3a) and completement 5a (C5a) were investigated. In terms of platelet, the activation and aggregation of platelet were determined. Based on above results, the interplay among coagulation system, complement and platelet incubating with ADs was proposed. Finally, the antioxidation of ADs was determined using the hemoglobin oxidation model, in which the effect of MW on iron chelation was discussed (Figure 1C).

## Materials and Methods

### Materials

Deferoxamine and sodium alginate were purchased from Sigma-Aldrich (St. Louis, MO, United States). NaBH_3_-CN and NaBH_4_ were obtained from AAD-50din Co. (Shanghai, China). Normal saline (NS), phosphate buffer saline (PBS), sodium periodate, peroxide hydrogen, polyetherimide and ferric sulfate, were obtained from Kelong Co., Ltd. (Chengdu, China). Heparin sodium was purchased from Alading Co., Ltd. (Chengdu, China). Blood was collected from Chengdu Blood Center. Coagulation-related kits were purchased from Union Bio-tech Co., Ltd. (Chengdu, China). The ELISA Kit II was purchased from Becton-Dickinson Co., Ltd. (United States). Flow cytometry-related agents, anti-CD61-fluorecein isothiocyanate (FITC), anti-CD62p-phycoerythrin (PE) and IgG1 (mouse) –PE, were purchased from BD Pharmingen, BD Bioscience Co., Ltd. (San Jose’, CA, United States). Platelet aggregation-inducers, adenosine-disphosphate and epinephrine, were obtained from Kelong Co., Ltd. (Chengdu, China).

### Synthesis of the ADs

The ADs were synthesized by Schiff-base reaction through the terminal amine groups in DFO and reactive aldehyde groups in oxidized alginate (OA) and followed by reduction as our previous report (Tian et al., 2016b). In brief, 10.0 g of sodium alginate was dissolved with 700 mL deionized water (DW) and 100 mL ethanol followed by different ratios of sodium periodate to uronate units (15, 30, 50 mol%) were used. The mixture was stirred in dark for 6 h and then mixed with 20 mL ethylene glycol before stirred for another 2 h. After that, to separate and purify each prepared ADA, the precipitant, presented by adding 10 g of NaCl and 2000 mL ethanol, was redissolved in 400 mL DW and 2000 mL ethanol again, and the progress was repeated three times. The products prepared from 15, 30, and 50 mol % of periodate/uronate units are corresponding to OA-15, OA-30, and OA-50 respectively. To obtain AD conjugates, 1 g of each OA was incubated with 2.5 g of DFO under stirring in room temperature. After 2 h, NaBH_3_CN (125 mg/mL) was slowly added to the solution under stirring for 4 h and then NaBH_4_ was added to the mixture for another 24 h. It respectively takes 3 days to dialyze against 1 M NaCl and DW using a dialysis tube (MWCO, 3500), totally 6 days. The conjugates were synthesized by OA-15, OA-30, and OA-50 named AD-15, AD-30, and AD-50 respectively. The structure of the synthesized conjugates was characterized as our previous report and shown in Table 1. The degree of DFO (% DFO) incorporation was calculated and described as moles of DFO attached per uronate residue.

**TABLE 1:** The structure characteristics of the conjugates.

**Conjugate**	**Mw (× 10^5^Da)**	**Mn (× 10^4^ Da)**	**PDI**	**DFO content (mol %)**
AD-15	2.5	4.9	5.0	8.7
AD-30	1.3	3.6	3.6	14.7
AD-50	0.6	1.8	3.5	20.4

### Blood Collection and Preparation of AD Solutions

The study was approved by Ethical Committee of Institute of Blood Transfusion, Chinese Academy of Medical Sciences and Peking Union Medical College. We collected blood samples at Chengdu Blood Center from three healthy donors. The whole blood was obtained through venipuncture and then mixed with 3.8% sodium citrate at radio of 9:1(blood/sodium citrate) to obtain citrated whole blood (CWB). To prepare 10% hematocrit of RBCs suspension, CWB was used to mix with the same volume of NS and centrifuged at 5000 r/min for 4 min, and then removed the supernatant. Repeated wash three times by NS for a total of four washes. While the supernatant was clear, removed it out and then added corresponding volume of NS to ensure the hematocrit of RBC suspension was 10%. CWB was centrifuged at 1200 *g* for 20 min to obtain platelet-rich plasma (PRP). Serum was prepared by centrifuging CWB he citrated at 1200 *g* for 30 min. The fresh frozen plasma (FFP) supplied by Chengdu blood center has anticoagulated with citrate-phosphate-adenine. ADs were dissolved in normal saline (NS) to obtain solutions with different concentration.

### Red Blood Cells

#### Hemolysis

The percent of RBC lysis was measured by the Free Hemoglobin Colorimetric Assay Kit using. Two hundred and seventy μl of RBC suspension (hematocrit: 10%) was respectively incubated with 30 μl of three ADs solutions with different concentrations for 1 h at 37°C to obtain a final concentration of 1, 5, and 10 mg/ml. We investigated the concentration of free hemoglobin by the method of ortho-tolidine to determine the hemolysis of incubated RBCs according to previous study (Lin et al., 2019). The absorbance was obtained by spectrophotometer (EON, Bio-Tech CO., Ltd., United States) at 435 nm. The percent of hemolysis was calculated by the following eq. (1). The RBCs suspension incubated with distilled water (DW) was used as a positive control.

(1) $$\mathrm{Hemolysis}\left(\%\right)=\frac{\text{A}\,\left(1\right)}{\text{A}\,\left(0\right)}\times \frac{100-\mathrm{Hct}\left(\%\right)}{\mathrm{Hb}\,\left(g/l\right)\times 1000}\times 100\%$$

A_(1)_: the absorbance of sample. A_(0)_: the absorbance of standard sample. Hct, hematocrit. Hb, 100 mg/L

#### Aggregation

Two hundred and seventy μl of CWB was respectively incubated with 30 μl of three ADs solutions with different concentrations for 1 h at 37°C to obtain a final concentration of 1, 5, and 10 mg/ml followed by centrifuging at 1000 *g* for 3 min. Three μl of RBCs sediment and 40 μl of supernatant were mixed and examined by optical microscopy. We captured the imagines by a digital microscope camera. CWB incubated with NS and polyetherimide (PEI) were respectively used as a normal control and positive control.

#### Deformability

Two hundred and seventy μl of CWB was respectively incubated with 30 μl of three ADs solutions with different concentrations for 1 h at 37°C to obtain a final concentration of 1, 5, and 10 mg/ml After incubation, the mixture (20 μl) was suspended in PBS (1 mL) containing 15% polyvinylpyrrolidone and then was tested by a Laser-diffraction Ektacytometer (LBYBX, Beijing Pencil Instrument CO., Ltd., China) according to the manufacture’s manual. Four shear stress, 0.39, 0.77, 1.54, and 7.7 Pa, were used, corresponding to the shear rate 50, 100, 200, and 1000 respectively. CWB incubated with NS was used as a normal control.

### Blood Coagulation

Sixty μl of three ADs solutions at 10 and 50 mg/ml were respectively incubated with 540 μl of FFP at 37°C for 3 min. After incubation, we used Prothrombin Time Diagnostic Kit to determine PT in the presence of Ca^2+^, and Activated Partial Thromboplastin Time Diagnostic Kit to determine APTT by adding kaolin and encephalin. We tested TT using Thrombin Time Determination Kit and the concentration of Fib in the presence of thrombin using Fibrinogen Determination Reagent Kit respectively. The coagulation time of incubated FFP, including APTT, prothrombin time (PT), thrombin time (TT), and the concentration of fibrinogen (Fib) were determined by an automated coagulation analyzer (Instrumentation Laboratory ACL ELITE, United States). To be specific, FFP incubated with NS and heparin (HP, 2 IU/mL) were respectively used as normal control and negative control.

### Complement Activation

The concentration of C3a and C5a were tested to prove the effect of ADs on complement system. Thirty μl of three ADs solutions at 10 and 50 mg/ml were respectively incubated with 270 μl of serum at 37°C for 3 min followed by analysis of complement activation using ELISA Kit II (Becton-Dickinson Co., Ltd., United States). The standards and samples were finally read by spectrophotometer (EON, Bio-Tech Co., Ltd., United States) at 450 nm and the concentration of C3a and C5a were calculated by utilizing standard curve. Serum incubated with zymosan (7 mg/ml) was used as a positive control.

### Platelet

#### Platelet Activation

Thirty μl of three ADs solutions at 10 and 50 mg/ml were respectively incubated with 270 μl of PRP at 37°C for 1 h. Five μl of incubated PRP, 5 μl of CD61, 5 μl of CD62p and 40 μl of PBS buffer (10 mM) were mixed in dark at 15 min and then added to 400 μl PBS (10 mM) for flow cytometric analysis. The activation of platelet was defined as the percentage of marker CD62p detected in 10000 total events counted by flow cytometry (Becton-Dickinson) using anti-CD61-fluorecein isothiocyanate (FITC), anti-CD62p-phycoerythrin (PE) and IgG1 (mouse)-PE (BD Pharmingen, BD Bioscience, San Jose’, CA, United States). The data was analyzed by BD FACSD via Software (Version 8.0.1.1). Human thrombin (10 IU/mL) and NS were respectively used as positive and normal control.

#### Platelet Aggregation

Thirty μl of three ADs solutions at 10 and 50 mg/ml were respectively incubated with 270 μl of PRP at 37°C for 1 h. Incubated PRP (225 μl) were mixed with two aggregation-inducers, adinosinedisphosphate (0.1 mM, 12.5 μl) and epinephrine (0.15 mM, 12.5 μl), and then we used platelet aggregometer (MODEL700, Chrono-Log Co., Ltd., United States) to determine the platelet aggregation.

### Antioxidation

To prove the effect of ADs on prevention of iron-mediated oxidation in the presence of hemoglobin (Hb), we observed the dynamic changes of oxyhemoglobin (oxyHb) while mixing ADs with ferric solutions containing Hb. Briefly, washed RBCs were frozen and thawed and then mixed with NS to obtain hemolysate (22 μM). To guarantee each sample, except for Hb control, contains 0.4 mM of DFO equivalent and 0.4 mM of ferric ion, ADs or DFO solutions (10 μl) and ferric sulfate solution (50 μl) were mixed with hemolysate solution (440 μl). The full wavelength scanning of each group was immediately performed by spectrophotometer (EON, Bio-Tech Co., Ltd., United States). In addition, the absorbance at 560, 576, 630, and 700 nm was respectively measured again every 30 s, in total of 5 min. The containing of oxyHbA was respectively calculated using the method of Winterbourn in different times, shown as the following eq. (2) (Winterbourn, 1985). The percent of oxyHbA was the ratio of oxyHbA to the primary containing of oxyHbA in blood samples.

$$oxyHbA\left(\%\right)=\frac{1.013\times \left(A\,1-\mathrm{A4}\right)-0.3269\times \left(A\,3-\mathrm{A4}\right)-0.7353\times \left(A\,2-\mathrm{A4}\right)}{10000}\times 100\%$$

A1 means the absorbance at 560 nm; A2 means the absorbance at 570 nm; A3 means the absorbance at 630 nm; A4 means the absorbance at 700 nm.

Further assessing the antioxidation activity of three AD conjugates and DFO, the degree of ADs and DFO chelating to iron was investigated. Fifty μl ferric sulfate solution (0.11 mM) was mixed with 50 μl AD conjugates or DFO solutions (DFO equivalent, 0.2 mM), and then the absorbance at 430 nm was respectively measured every 5 s, in total of 1 min.

### Statistical Analysis

We use analysis of variance and paired *t*-test to perform the statistical analysis. The probability (*P*) values less than 0.05 was considered to have significant difference and was calculated with the software assistance of Excel 2007 and SPSS 19.0. The results are presented as mean ± SD (*n* = 3).

## Results and Discussion

### Synthesis of ADs

ADs were synthesized through a two-step process in which alginate was oxidized to ADA firstly, and then the conjugates were prepared by Schiff-base reaction through the terminal amine groups in DFO and reactive aldehyde groups in ADA and followed by reduction with NaBH_3_CN and NaBH_4_ (Figure 1A). To prepare a series of conjugates with different molecular weight, alginate was oxidized to different oxidation degree by adjusting the mol ratio of periodate/uronate units, since oxidation of alginate with sodium periodate results not only cleavage of the C2-C3 bond, but also main chain scission as a simultaneous reaction. The structure characteristics of the prepared conjugates are summarized in Table 1. As expected, the Mw of the conjugates are 245, 128, and 62.7 kDa, respectively. The DFO contents in the conjugates were increased with the decrease of the Mw due to the coupling of the oxidation and Mw, with 8.7, 14.7, and 20.4% by molar and corresponding to around one DFO per eleven, seven, and five uronate units for AD-15, AD-30, and AD-50, respectively.

### Red Blood Cells

**FIGURE 2:**
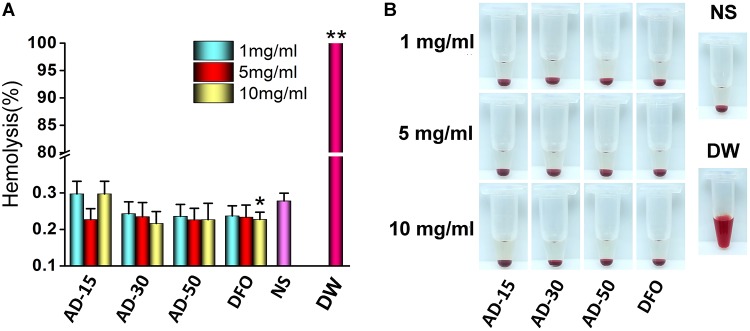
Effect of ADs on hemolysis. Three ADs and DFO were incubated with 10% hematocrit RBCs suspension at 1, 5, and 10 mg/mL. **(A)** The percent of hemolysis, **(B)** the visual appearance of AD, DFO and controls. *P < 0.05 versus NS; **DW has significant differences with the others, *P* < 0.05.

Hemolysis is characterized as the rupture of RBCs and the release of the cytoplasm, and the evaluation of hemolysis is essential regarding the biosafety of exogenous materials. To investigate the device-associated hemolysis, static or semi-static testing *in vitro* was widely used (Haishima et al., 2014). Based on ISO10993-5, ISO10993-4 and ASTMF756-00, the percent less than 5% will be considered as a very low risk of hemolysis (Zhen et al., 2015). According to the results as shown in Figure 2, the percent of hemolysis in AD-15, AD-30, AD-50 and DFO at any concentration fluctuated around 0.2%, while the hemolysis of DW group is approximately 90%. Except for the DFO in 10 mg/mL, the others rarely showed any significant difference compared with normal saline (NS), indicating ADs had a quite low risk of hemolysis.

**FIGURE 3:**
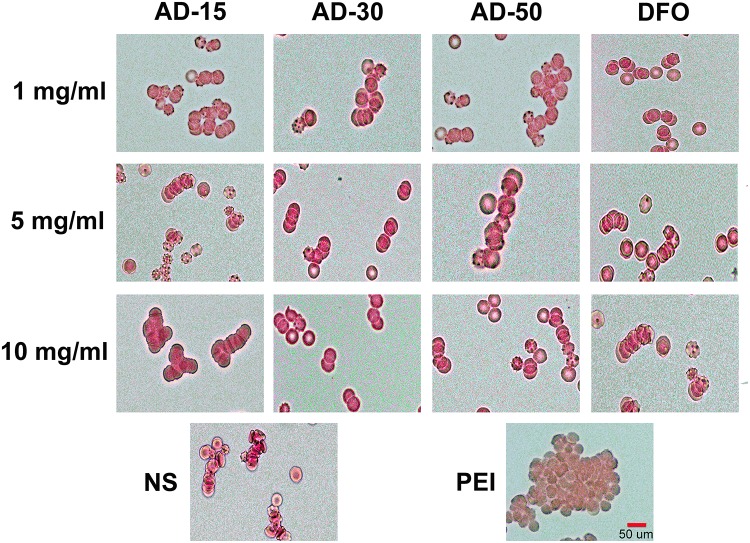
Red blood cells aggregation. Three ADs and DFO were incubated with whole blood at 1, 5, and 10 mg/mL. The RBCs in plasma was observed by a digital microscopy followed by capturing the imagines.

The property of RBCs to aggregate is one of the typical features that plays a vital role in the blood circulation (Claveria et al., 2016). The aggregation of RBCs has been proved to affect their viscosity ability owing to the non-Newtonian behavior of blood in the body and reversible aggregation may physiologically occur in the presence of macromolecules and calcium ion, serving as the bridge among RBCs (Mehri et al., 2014). However, irreversible RBCs aggregation, induced by some chemicals, such as phosphodiesterase and polyethyleneimine (PEI), could damage RBCs and even potentially disrupt capillaries (Muravyov and Tikhomirova, 2014). Herein, photomicrographs of RBCs incubated with PEI, used as positive control, and ADs at 1, 5, and 10 mg/mL were shown in Figure 3. Compared with PEI, neither ADs nor DFO showed any irreversible aggregation. The likely aggregation in ADs group as well as NS control was contributed to rouleau formation, as a typical result of reversible aggregation (Flormann et al., 2015). According to the previous study, it has long been known that alginate increased whole blood viscosity and induced aggregation of RBCs thus being used as a viscosity modifier for blood substitutes (Xu et al., 2017). In this work, ADs showed negative response to RBCs aggregation, which could be ascribed to the possibly low content of alginate in the reaction system (Zhao et al., 2010).

**FIGURE 4:**
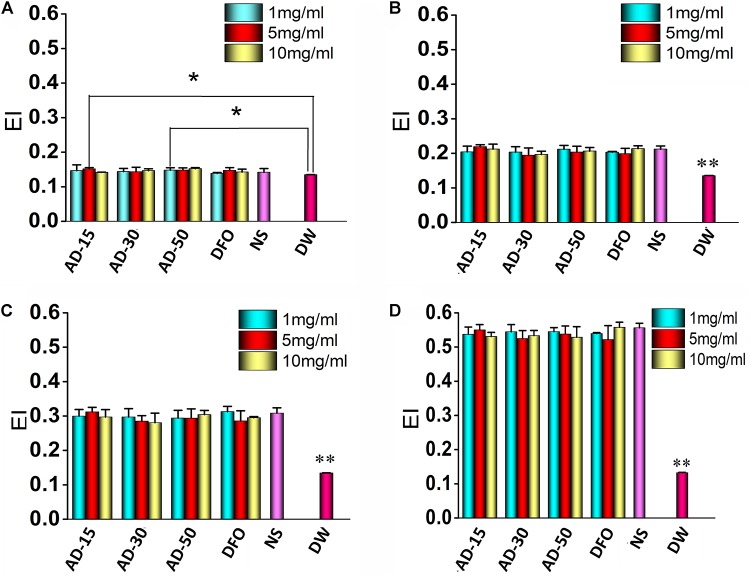
Red blood cells deformability. Three ADs and DFO were incubated with whole blood at 1, 5, and 10 mg/mL and then, incubated RBCs were tested by a Laser-diffraction Ektacytometer at different shear stress, **(A)** 0.39, **(B)** 0.77, **(C)** 1.54, and **(D)** 7.7 Pa. *P < 0.05 versus DW; **DW has significant differences with the others, *P* < 0.05.

The property of allows them to change their shape according to the diameter of blood vessel as a result of natural shape of RBCs. The regulation of RBCs deformability depends on three aspects, cytoplasm viscosity, membrane mechanical properties and surface area and volume of RBCs (Uhl et al., 2018). Malfunction of RBCs deformability is contributed to the occurrence of several diseases, such as sickle cell anemia, malaria and hereditary spherocytosis (Zhao et al., 2010). Figures 4A–D showed the deformability of incubated RBCs at various shear stress. The results indicated there is no significant difference among three ADs and NS at any concentration or shear stress. The deformability of DW group was significantly lower than three ADs at the shear rate 100, 200, and 1000 while at 50, DW group was slightly lower than AD-15 at 5 mg/ml (P = 0.046) and AD-30 at 1 mg/ml (*P* = 0.038). Generally, the elongation index (EI) significantly increased while being exposed to a higher shear stress. For DW group, the EI stayed a low level as the shear rate changed, suggesting that the deformability of RBCs could be significantly suppressed in the presence of hemolysis.

### Coagulation Function

**FIGURE 5:**
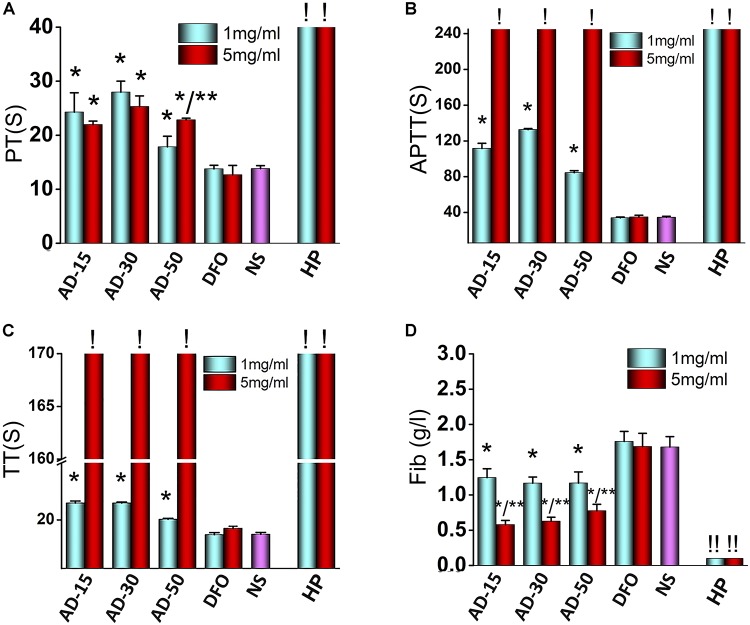
Effect of ADs on blood coagulation. Three ADs and DFO were incubated with fresh frozen plasma (FFP) at 1 and 5 mg/mL. **(A)** PT, **(B)** APTT, **(C)** TT, and **(D)** Fib, were tested by an automated coagulation analyzer. *P < 0.05 versus NS or DFO; **means 5 mg/mL has significant differences with 1 mg/mL, *P* < 0.05. **!** : The values are too high that cannot be determined by the analyzer (APTT > 245 s, TT > 169 s). **! !**: The values are too low that cannot be determined by the analyzer. HP, heparin.

To determine the effect of ADs on coagulation system, we tested the coagulation time, including PT, APTT, and TT, together along with the concentration of Fib. The results of blood coagulation were shown in Figure 5. Comparing with DFO or NS group, the PT of three ADs, on behalf of extrinsic coagulation pathway, were simultaneously lengthened to some extent. Unlike AD-15 and AD-30, the PT of AD-50 presented a dose-dependent effect. For APTT and TT, reflecting the intrinsic coagulation pathway and common coagulation pathway respectively, three ADs significantly prolonged APTT and TT compared with DFO or NS in a dose-dependent manner and at the concentration of 5 mg/mL, the values of APTT and TT were too high to be tested by analyzer (the detection range for APTT is 6–245 s, for TT is from 3–169 s). In terms of Fib, the concentration of Fib of three ADs was approximately 1.2 g/L while DFO or NS group was approximately 1.6 g/L. Additionally, ADs could decrease the concentration of Fib in a dose-dependent manner since the concentration of Fib ADs at 5 mg/mL was approximately 0.6 g/L with statistical difference.

To the best of our knowledge, this is the first study demonstrated that polymer-drug conjugate using alginate as drug carrier was capable of anticoagulant activity. ADs significantly prolonged the coagulation time, particularly APTT, indicating that the intrinsic pathway was dominantly blocked in the process of anticoagulation. To explain this phenomenon, two main mechanisms were proposed to involve in anticoagulation pathway. Firstly, interacting with the coagulation factors, such as factor I (Fib), II, V, and X, ADs can significantly inhibit their activity, thus preventing them from participating in coagulation cascade reaction (Zou et al., 2014; Xin et al., 2016). Secondly, the anticoagulative activity relates with the negative functional groups along the chains of the polysaccharides since the negative functional group would form a complex interaction with antithrombin in plasma (Li et al., 2017). Alginate is the only polysaccharide that naturally contains negative carboxyl groups along the chains, indicating that anticoagulant activity probably has to be involved. Besides carboxyl groups, there are many hydroxyl groups along the chains of alginate, both of which have been suggested to have significant effect on the coagulation function (Shiu et al., 2014; Guo et al., 2018). The anticoagulation induced by carboxyl groups and hydroxyl groups is supported by the work of Sperling et al. (2005), who studied the influence of different content of carboxyl groups or hydroxyl groups on coagulation function and indicated that pure-carboxyl groups showed a strong anticoagulative effect while the effect of pure-hydroxyl groups was milder.

Among three ADs, AD-50, with the lowest MW, showed a weaker anticoagulative activity than that of AD-15 and 30, indicating that ADs-induced anticoagulation was partially depended on the MW, which was consistent with previous studies. For instance, Xu et al. (2017) studied the blood compatibility of alginate with different MW (1170–50075 kDa) prepared by heterogeneous phase acid degradation and the results indicated that the blood clotting time was prolonged with increasing of MW of alginate. Similarly, the anticoagulation of sulfated polysaccharides depends not only on the substituted functional groups along main chains, but also the MW. Xin et al. (2016) investigated the anticoagulative properties of a series of low MW propylene glycol alginate sodium sulfate (2.99–8.91 KDa) and they found it could prolong the APTT and clotting time and the anticoagulative activity declined with the decrease in MW. Besides, Fan et al. (2011) aimed at prove the correlation between MW and anticoagulative activity and indicated that the clotting time induced by sodium alginate sulfate (14900–35300 KDa) initially increased as the MW decreased but fall with the further decrease in MW. Therefore, based on the previous study, it is suggested that within a certain MW range, the degree of anticoagulative activity through absorption coagulation factors through negative charged groups along the chains has a main dependence on MW, and with the reduction of MW, the anticoagulation activity is much weaker probably ascribing to the decrease of negative groups.

In addition, it should be noted that the anticoagulative effect induced by ADs were weaker than HP, a widely used anticoagulant in clinic. Besides, the mild anticoagulative activity of ADs would rather extend the application in the treatment of iron-overload diseases. For instance, in some iron-overload diseases like thalassanemia and sideroblastic anaemia, long-term repeated-transfusion is needed to improve the content of hemoglobin while transfusion-associated thrombosis is unacceptably common in clinic as well (Xenos et al., 2012). To simultaneously overcome iron-overload and hypocoagulability state, ADs show superiorities contributing to their anticoagulative effect. Additionally, hemorrhage is another concern in clinic following the application of anticoagulant and some research indicated that the concentration of Fib had a closely link with the incidence of hemorrhage and there is a much higher risk of bleeding as Fib concentration is less than 0.5 g/L (Lunde et al., 2014). In this light, our results showed that the concentration of Fib of three ADs at 1 or 5 mg/mL were higher than HP group, indicating that ADs had a quite lower risk of hemorrhage than HP.

### Complement Activation

**FIGURE 6:**
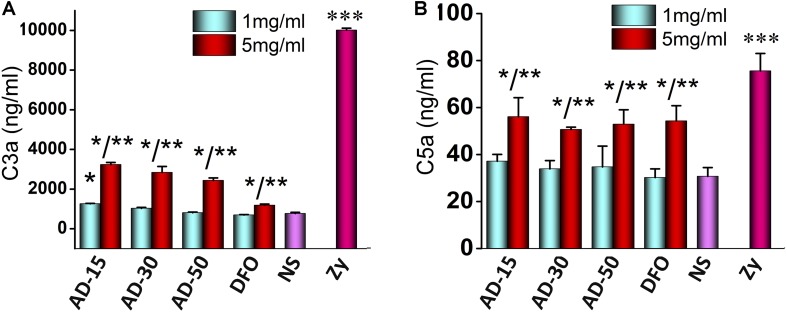
Effect of ADs on complement activation. Three ADs and DFO were incubated with serum at 1 and 5 mg/mL and then, **(A)** C3a and **(B)** C5a were investigated by ELISA KIT II. *P < 0.05 for NS; **5 mg/mL has significant differences with 1 mg/mL, *P* < 0.05; ***P < 0.05 for the others. zy, zymosan.

Complement can be activated by cascade reaction in three pathway, classic pathway, alternative pathway and mannose binding lectin pathway (Koscielska-Kasprzak et al., 2014). It has long been known that exogenous biomaterials mainly activate alternative pathway, which could cause severe side-effects, such as inflammation (Liu et al., 2013). In alternative pathway, C3, directly activated by biomaterials, cleaves to produce C3a and C3b. C3b can regulate the function of monocytes and macrophages. C3a is component of C3ADesArg, and make up C5 convertase, which causes change of C5 to C5a and C5b. C5a and C5b are component of C5ADesArg and membrane attack complex respectively. In a word, concentrations of C3a and C5a are canonical indexes to access the activation of complement pathway (Nesargikar et al., 2012). Serum incubated with 1 and 5mg/mL AD conjugates or DFO were adopted to measure the concentrations of C3a and C5a. The results of incubated complement were shown in Figures 6A,B. At the concentration of 1 mg/mL, except for the mild activation of C3 induced by AD-15, C3 and C5 were rarely activated by AD-30 or AD-50. At the concentration of 5 mg/mL, C3a and C5a of three ADs sharply increased almost two times than low concentration suggesting high-dose of ADs could activate complement system through alternative pathway. The activation of C3a and C5a induced by ADs, however, were significantly lower than that of positive control, zymosan. The dose-dependent complement activation is closely dependent on the content of hydroxyl groups along the chains, one of negative groups that can trigger the complement cascade response (Zhong et al., 2013). Besides binding to complement directly, hydroxyl group also interact with complement through absorption numerous complement-related proteins (Wetterö et al., 2002).

### Platelet

**FIGURE 7:**
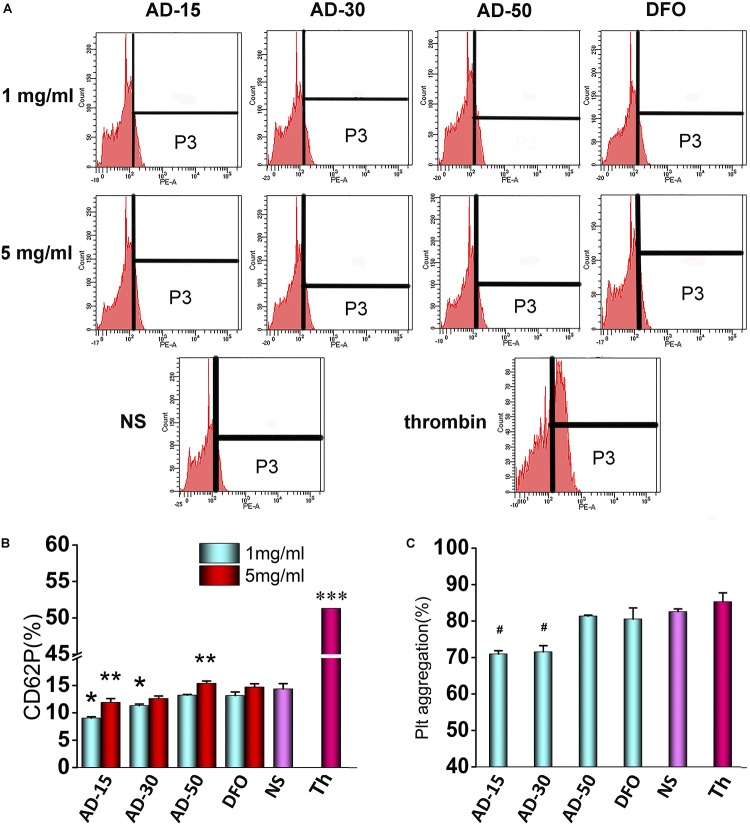
Effect of ADs on platelet. **(A)** Flow cytometry data profiles from platelet activation analysis. The part of P3 means the percent of CD62P. **(B)** Platelet activation. Three ADs and DFO were incubated with platelet-fresh plasma (PFP) at 1 and 5 mg/mL for and the expression of CD62P was determined using anti-CD62P antibody by flow cytometry. **(C)** Platelet aggregation. Three ADs and DFO were incubated with platelet-fresh plasma (PFP) at 1 mg/mL and then, the aggregation was determined by aggregometer. *P < 0.05 for NS; **5 mg/mL has significant differences with 1 mg/mL, *P* < 0.05; ***P < 0.05 for the others; #*P* < 0.05 versus NS and thrombin. Th, thrombin.

Platelets, derived from megakaryocytes in the bone marrow with plasmolemma, usually maintain the integrity of blood vessel and participate in the process of hemostasis following vascular injury (Sun et al., 2019). CD62P (platelet surface *P*-selectin) has long been used as markers to determine the activation of platelet since its considerable stability (Lu and Malinauskas, 2011). In this work, CD62P was used to determine the effect of ADs on platelet activation using flow cytometry. To further measure platelet function, the aggregation of incubated platelet was investigated using platelet aggregometer. The expression of CD62P and the percent of platelet aggregation were shown in Figure 7. At the concentration of 1 mg/mL, AD-15 and AD-30 slightly suppressed platelet activation compared with NS, which is accordant with the results of platelet aggregation. Compared with ADs at 1 mg/mL, mild activation of platelet can be observed at the concentration of 5 mg/mL. Based on the results, AD-50 rarely influenced the platelet function.

**FIGURE 8:**
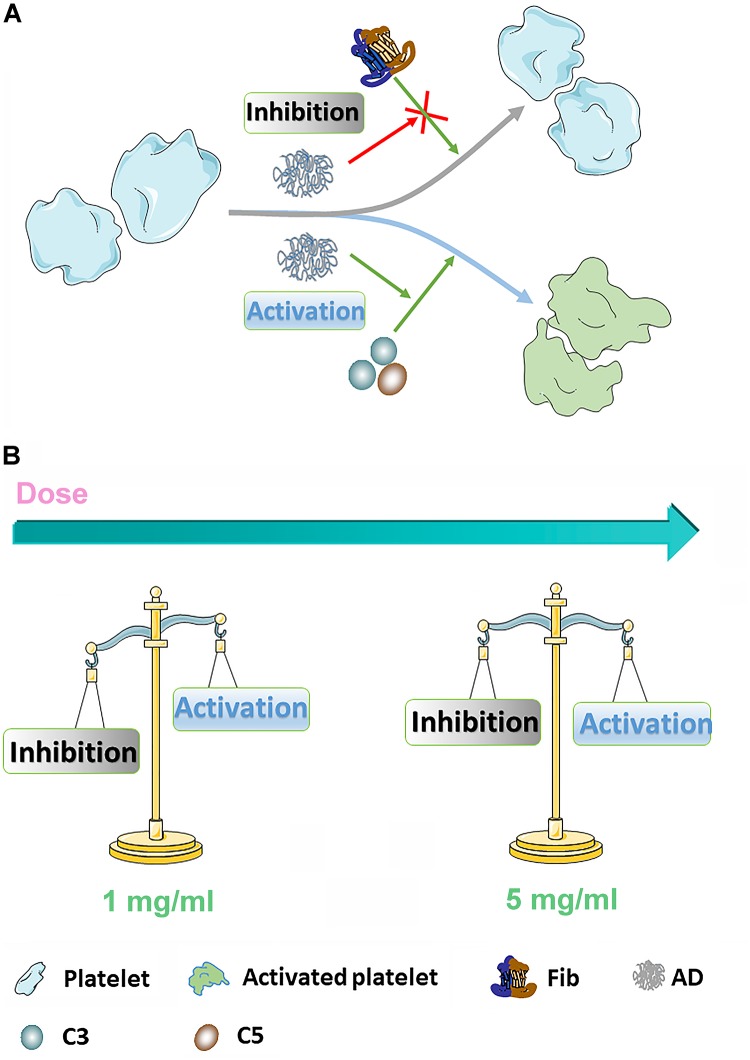
**(A)** The diagrammatic sketch of coagulation- and complement-associated platelet activation. ADs can inhibit platelet through absorbing Fib, serving as an important stimulator for platelet activation. On the other hand, AD conjugates can also stimulate platelet through activating complement system. **(B)** In 1 mg/mL, coagulation-associated inhibition is dominant leading to a reduction of platelet activation for AD-50 and AD-30 while in 5 mg/mL, the coagulation-associated inhibition and complement-associated activation keep in balance.

Platelet activation, in which fib has been reported one of requisite stimulating factors, involving in a complicated multistep process including adhesion, release and aggregation for hemostatic plug formation and thrombosis, has been reported that could be influenced by coagulation function and complement system in the blood (Khanbeigi et al., 2015). For instance, coagulants, like thrombin, can significantly activate platelet and inhibit the coagulation process while some complement inhibitors can similarly inhibit the platelet function (Speth et al., 2015). However, few studies fully revealed the interplay of coagulation and complement in platelet function *in vitro*. Therefore, according to our findings, it is proposed that AD conjugates can inhibit the platelet by the means of inhibition of Fib through functional groups along the chains and in the meantime, the inhibition can be antagonized by complement activation to some extent. However, few studies fully revealed the interplay of coagulation and complement in platelet function *in vitro*. According to the results of coagulation, complement, and platelet function, it is proposed that ADs can inhibit the platelet by the means of inhibition of Fib through functional groups along the chains, which could be antagonized by complement-associated activation to some extent. As shown in Figure 8, ADs, as mild anticoagulants, can absorb Fib to make them be absence of the process of platelet activation through the functional groups along the chains, like hydroxyl and carboxyl group. However, with the elimination of anticoagulative activity, the anticoagulation-associated platelet inhibition is negligible since AD-50 barely influenced the platelet activation. On the other hand, it has been reported that platelets express many complement molecules, including some complement receptors and complement regulatory molecules that can generate C3a and C5a and as a return, the complement system and its activation products can also stimulate platelets by several complement factors, such as the anaphylatoxins C3a and C5a (Speth et al., 2015). Herein, at the concentration of 1 mg/mL, comparing to the platelet inhibition through absorbing Fib, complement-associated activation induced by ADs is milder as a result of slight reduction on platelet activation. However, at the concentration of 5 mg/mL, with the raised activity in both anticoagulation and complement, the complement-induced activation is significantly enhanced against the anticoagulation-associated inhibition, being balanced and resulting in equal level of platelet activation between ADs and NS.

### Antioxidation

**FIGURE 9:**
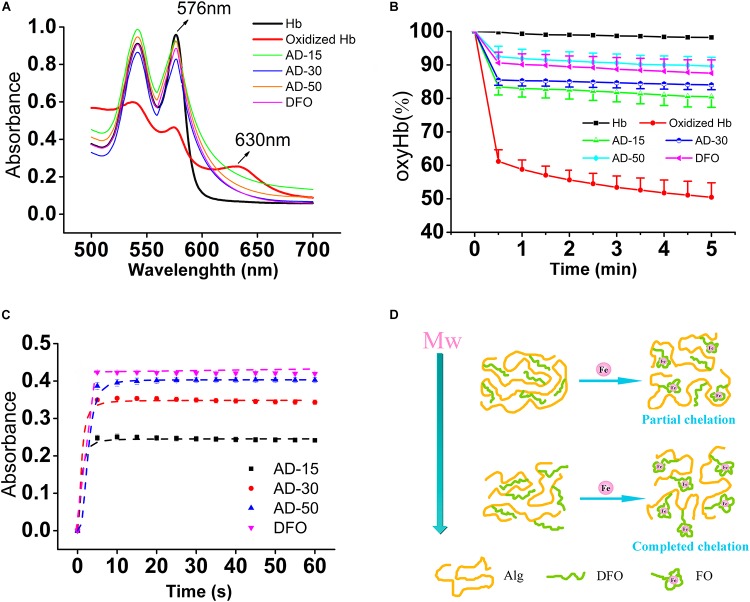
Protection against iron-mediated oxidation in hemolysate. Each group, except for the hemolysate (Hb) and oxyHb (containing Hb and Fe^3+^) control, similarly contains 0.4 mM of DFO equivalent (AD or DFO) and Fe^3+^, dissolving in Hb solution (22 μM). **(A)** The full wavelength scanning. **(B)** The tendency of the percent of oxyHb in each group, scanning every 30 s in total of 5 min. **(C)** The lever of DFO-Fe^3+^ chelates. The absorbance was obtained at 430 nm, which means the degree of ADs or DFO chelating to iron. **(D)** The diagrammatic sketch of different MW ADs to Fe^3+^. With the Mw of AD conjugates increasing, the ability to chelating to iron will be repressed ascribing to the steric hinderance effect.

To prove the prevention of iron-mediated oxidation, hemoglobin (Hb) oxidation model, in which mimic the process underlying the pathology of hemoglobinopathies such as sickle cell anemia and the thalassemia, was widely used (Xu et al., 2014). Under normal condition, the Hb converts to oxyHb in ferrous state (Fe^2+^), able to bind and transport oxygen, and methemoglobin in the presence of ferric iron (Fe^3+^) (Pichert and Arnhold, 2015). As shown in Figure 9A, oxyHb has two obvious peaks between 500 and 600 nm while methemoglobin (oxidized Hb) has another new peak at 630 nm, a feature that can be utilized to distinguish these two proteins. Upon addition of DFO, the absorption spectrum that refers to content of oxyHb is similar to oxyHb rather than oxidized Hb control, suggesting that the conjugates can bind to ferric iron to protect Hb from being oxidized to methemoglobin in Fe^3+^-rich condition. To detail the prevention, the percent of oxyHb was calculated on the basis of absorbance at 560, 576, 630, and 700 nm. The results are shown in Figure 9B. The content of oxyHb in ADs and DFO are dramatically higher than oxidized Hb control (containing Hb and Fe^3+^) all the time, suggesting that ADs or DFO as a chelator for ferric iron prevents Hb from being oxidized. However, the binding to iron could be time-dependent since a sharply reduction is observed in the first 30 s, and then reaches a stable lever. In terms of three ADs, additionally, AD-50 seems to exhibit a slightly better capability on antioxidation comparing to AD-15 and AD-30. Therefore, the ability of the conjugates and the free DFO binding to Fe^3+^ were further determined by spectrophotometer and the results are shown in Figure 9C. The degree of ADs or DFO chelating Fe^3+^ rapidly reach to the peak in the first 5 s, which means the binding to Fe^3+^ occurs as soon as they contact. For three ADs with similar DFO content, the level of chelates significantly augment as the MW reduction while the free of DFO is slightly higher than that of conjugates. As a result, it is indicated that the ADs are capable of antioxidant activity mainly through iron chelation in a MW dependence manner.

Although there are several reports attempt to reveal the links between MW and antioxidative property, that found biomaterials with a lower MW have a higher antioxidant activity, the mechanism remains controversial. Alginate has its intrinsic antioxidant activity due to the reductive ability of residues along the molecular chains and its antioxidant activity is increased with the decrease of the MW (Xu et al., 2017), which may benefit to protect Hb from oxidation in a different manner comparing to the free of DFO. However, the conjugates did not show superior antioxidant activity as the expected. The reasons for the MW dependence of the iron chelation probably relate with the polymer carrier-induced steric hinderance. By determining the scavenging ability of 1, 1-diphenyl-2- picrylhydrazyl free radical (DPPH), alginate with a lower MW shows a better antioxidant activity probably ascribe to the increase in the a-L-guluronic acids (G) content of alginate and in the meanwhile, the nature of alginate chain was extended due to the diaxial linkage in G-blocks, which may hind the rotation around glycosidic acid as the MW increase (Şen, 2011). Nevertheless, the polymer drug carrier also leads to steric hinderance for DFO chelating, resulting in iron binding occurring at a slower rate, depending on the density and location of conjugated DFO molecules (Banerjee, 2009; Yu et al., 2019). As shown in Figure 9D, iron chelation results in a locally ordered molecular configuration which is a process of losses of entropy. The higher MW and longer molecular chains, the higher losses of entropy, which in turn makes it more difficult for iron chelation.

## Conclusion

In conclusion, we successfully synthesized a series of ADs with various MW and the interactions with RBCs, coagulation, complement, and platelet had been studied for the conjugates as a function of their dose and MW. Interactions of ADs with RBCs did not reveal any hemolysis and showed reversible aggregation contributing to rouleau formation of RBCs and normal deformability ability. On the contrary, ADs significantly prolonged the coagulation time in a dose-dependent manner, particularly APTT and TT, suggesting ADs could dominantly inhibit the intrinsic and common pathways in the process of coagulation. The results of complement and platelet tests showed ADs could activate complements C3a and C5a, presenting dose-dependence while AD-15 and AD-30 slightly inhibit the platelet activation and aggregation in low concentration. Besides, the interplay among coagulation, complement, and platelet activation was proposed. Finally, the antioxidant activity of ADs was demonstrated in a MW-dependent manner.

## Data Availability Statement

All datasets generated for this study are included in the article/supplementary material.

## Ethics Statement

The studies involving human participants were reviewed and approved by the Institute of Blood Transfusion, Chinese Academy of Medical Sciences and Peking Union Medical College. The patients/participants provided their written informed consent to participate in this study.

## Author Contributions

TS performed the blood evaluation, wrote the manuscript, and discussed the results. XG performed the blood evaluation. RZ performed the other experiments. CW, HLiu, HLi, LM, JG, and CY were involved in the results discussion. MT was responsible for conceptualization, results discussion, and revising the manuscript.

## Conflict of Interest

The authors declare that the research was conducted in the absence of any commercial or financial relationships that could be construed as a potential conflict of interest.
